# Modelling of Spray Flames with Doubly Conditional Moment Closure

**DOI:** 10.1007/s10494-017-9873-3

**Published:** 2017-11-20

**Authors:** Michael Philip Sitte, Epaminondas Mastorakos

**Affiliations:** Department of Engineering, University of Cambridge, Cambridge, CB2 1PZ UK

**Keywords:** Conditional moment closure, Spray flames, Pre-vaporisation, Ethanol

## Abstract

Simulations of a pilot-stabilised flame in a uniformly dispersed ethanol spray are performed using a Doubly Conditional Moment Closure (DCMC) model. The DCMC equation for spray combustion is derived, using the mixture fraction and the reaction progress variable as conditioning variables, including droplet evaporation and differential diffusion terms. A set of closure sub-models is suggested to allow for a first, preliminary application of the DCMC model to the test case presented here. In particular, the DCMC model is used to provide complete closure for the Favre-averaged spray terms in the mean and variance equations of the conditioning variables and the present test case is used to assess the importance of each term. Comparison with experimental data shows a promising overall agreement, whilst differences are related to modelling choices.

## Introduction

In a broad range of combustions devices, including most mobile applications such as aero-engines and IC-engines, fuel is supplied in liquid form. Modelling of spray flames is challenging, even when ignoring the difficulties related to dense sprays or the modelling of atomisation. In particular, the complex interplay of droplet evaporation, turbulent mixing and chemical reaction in the presence of large mixture inhomogeneities leads to a wide variety of spray combustion regimes and phenomena [1].

Many experimental and numerical studies have explored the propagation of flames through disperse sprays. Whilst flame propagation in a mist of very small droplets was similar to the case of a homogeneous mixture, larger droplets were found to have a positive effect on the burning velocity [2, 3]. Yet, an inverse correlation of burning velocity with Sauter mean diameter was found above a certain droplet size [4]. In a numerical study of flame propagation in quiescent sprays, Neophytou and Mastorakos [5] showed that the effective equivalence ratio, as compared to the overall equivalence ratio, was an important parameter with respect to the burning velocity. Direct numerical simulations (DNS) showed that the flame propagation consisted of the successive ignition of flames engulfing individual droplets [6] and that premixed and non-premixed combustion modes co-exist in spray flames [7–9].

In comparison to laminar flame propagating in a droplet mist, turbulent spray flames have been studied less. In turbulent combustion, there is an additional need of modelling to take turbulent-flame interaction into account appropriately. A short overview of the modelling approaches for turbulent spray flames can be found in the review by Jenny et al. [1]. In order to capture finite kinetic effects, pyrolysis and pollutant formation, an advanced combustion model is required.

Conditional Moment Closure (CMC) is a statistical model for turbulent combustion, making use of a strong correlation between the reacting scalars and the conditioning variables. It then provides a very simple closure for the highly non-linear reaction source term, whilst its derivation only requires very light assumptions on the physics involved, such that its application is *a priori* not limited to a specific combustion mode or regime [10, 11]. CMC was originally developed for non-premixed flames [10] but it has since been extended to spray combustion [12, 13] and its applicability to premixed flames has also been demonstrated [14, 15]. In all these models a single conditioning variable, either mixture fraction of reaction progress variable, is used. However, this was found insufficient in certain flames, e.g. in the presence of significant extinction, and Kronenburg [16] demonstrated the effectiveness of introducing a second conditioning variable to provide more accurate CMC closure of the reaction source term.

This wide range of successful applications makes CMC an attractive candidate for the modelling of flames that have both premixed and non-premixed features, in particular, by pursuing the strategy of double conditioning. The present work focuses on the development of a Doubly Conditional Moment Closure (DCMC) model for spray combustion. The objectives of this paper are to (i) present the DCMC equation for spray flames, (ii) provide closure by suggesting a set of sub-models, in order to (iii) apply the model in a first, preliminary test to a pilot stabilised flame in a uniformly dispersed ethanol spray and (iv) compare the simulation results with experimental data of this burner recently studied at the University of Cambridge [17]. This flame behaves as premixed due to pre-vaporisation but with the flame still generating its own vapour and hence provides a relevant test case.

## Methodology

### Derivation of the DCMC equation

In the Conditional Moment Closure combustion model, transport equations are solved for the conditional moments *Q*_*α*_(**Z** ^*c*^;**x**, *t*) ≡〈*Y*_*α*_(**x**, *t*)|**Y** ^*c*^(**x**, *t*) = **Z** ^*c*^〉 of the reactive scalars *Y*_*α*_, which are conditionally averaged on a subset of the space of all reactive scalars **Y** ^*c*^ ⊂**Y** [10]. The probability density function (pdf) of the conditioning variables is presumed and mean quantities of the reactive scalars are calculated by integrating the conditional moments with the pdf. In contrast to conventional CMC, in the present DCMC approach the reactive scalars are conditioned on two conditioning variables.

The DCMC model equation for spray flames is derived following the approach by Mortensen and Bilger [12] who used a separated flow model to incorporated the effects of spray evaporation. With a separated flow model the local instantaneous balance equations for a multi-phase flow are written by introducing a phase indicator function *𝜃*_*k*_(**x**, *t*), which is unity in the region occupied by phase *k* and zero everywhere else [18, 19]; its governing equation is as follows.

1 $$\begin{array}{@{}rcl@{}} \frac{\partial \theta_{k}}{\partial t} + \mathbf{u}_{k}\cdot\nabla\theta_{k} = \varPi_{k} \end{array} $$

Here π_*k*_ is the volumetric rate of phase change per unite volume. Then the continuity equation and the species transport for the phase *k* take the following form [12],

2 $$\begin{array}{@{}rcl@{}} \frac{\partial \theta_{k} \rho_{k}}{\partial t} + \text{div}(\theta_{k} \rho_{k} \mathbf{u}_{k} ) = \rho_{k} \varPi_{k} \end{array} $$

3 $$\begin{array}{@{}rcl@{}} \frac{\partial \theta_{k} \rho_{k} Y_{k,\alpha}}{\partial t} + \text{div}(\theta_{k} \rho_{k} Y_{k,\alpha} \mathbf{u}_{k} ) & = & \text{div}(\theta_{k} \rho_{k} D_{k,\alpha} \nabla Y_{k,\alpha}) + \theta_{k}\rho_{k}\dot{\omega}_{k,\alpha} \\ &&+ \rho_{k} Y_{k,\alpha} (\hat{V}_{k,\alpha} + \varPi_{k}) \end{array} $$

where $\hat {V}_{k,\alpha } \equiv \nabla \theta _{k} \cdot \mathbf {V}_{k,\alpha } = \nabla \theta _{k} \cdot (-D_{k,\alpha }/Y_{k,\alpha })\nabla Y_{k,\alpha }$ is the diffusion velocity across the phase interface. It can be related to π_*k*_ through the jump conditions at the interface [18, 19],

4 $$\begin{array}{@{}rcl@{}} \sum\limits_{k = 1}^{2} \rho_{k} \varPi_{k} = 0 \end{array} $$

5 $$\begin{array}{@{}rcl@{}} \sum\limits_{k = 1}^{2} \rho_{k} Y_{k,\alpha} \varPi_{k} + \rho_{k} Y_{k,\alpha} \hat{V}_{k,\alpha} = 0 \end{array} $$

In this work the subscript *k* may only refer either to the gas or to the liquid phase and is, therefore, omitted in further derivations.

The present DCMC approach uses the mixture fraction *ξ* and the reaction progress variable *c* as conditioning variables. Whilst the mixture fraction is a passive scalar with respect to chemical reaction, it is produced through droplet evaporation. It is defined to be 0 in air and 1 in undiluted fuel vapour. For a pure liquid fuel the jump condition at the phase interface (5) for the mixture fraction gives $\xi \hat {V}_{\xi }=(1-\xi )\varPi $ [12], which leads to the following transport equation of the mixture fraction,

6 $$\begin{array}{@{}rcl@{}} \frac{\partial \theta \rho \xi}{\partial t} + \text{div}(\theta \rho \xi \mathbf{u} ) = \text{div}(\theta \rho D_{\xi} \nabla \xi) + \rho\varPi \end{array} $$

The progress variable is defined as a linear expression of one reactive scalar *Y*_*ψ*_, normalised by its un-reacted and equilibrium values, $Y_{\psi }^{0}(\xi )$ and $Y_{\psi }^{\text {Eq}}(\xi )$ respectively [20, 21].

7 $$\begin{array}{@{}rcl@{}} c(\mathbf{x},t) = c_{\psi}(\xi(\mathbf{x},t), Y_{\psi}(\mathbf{x},t)) = \frac{Y_{\psi}^{0}(\xi(\mathbf{x},t)) - Y_{\psi}(\mathbf{x},t)}{Y_{\psi}^{0}(\xi(\mathbf{x},t)) - Y_{\psi}^{\text{Eq}}(\xi(\mathbf{x},t))} \end{array} $$

If *Y*_*ψ*_ is governed by an equation of the same form as Eq. 3, then the instantaneous transport equation of the reaction progress variable *c* = *c*_*ψ*_ is,

8 $$\begin{array}{@{}rcl@{}} \frac{\partial \theta \rho c}{\partial t} + \text{div}(\theta\rho c \mathbf{u} ) &=& \text{div}(\theta\rho D_{c} \nabla c)\\ &&+ \underbrace{\frac{\theta\rho }{\partial Y_{\psi} / \partial c} \left[\dot{\omega}_{\psi} + N_{\xi} \frac{\partial^{2} Y_{\psi}}{\partial \xi^{2}} + 2 N_{\xi c} \frac{\partial^{2} Y_{\psi}}{\partial \xi \partial c} + N_{c} \frac{\partial^{2} Y_{\psi}}{\partial c^{2}}\right]}_{\theta\rho\dot{\omega}_{c}^{*}} \\ &&+ \underbrace{ \frac{\rho }{\partial Y_{\psi} / \partial c} \left[Y_{\psi} \hat{V}_{\psi} - \xi\hat{V}_{\xi} \frac{\partial Y_{\psi}}{\partial \xi} \right] }_{\rho \mathscr{C}(\xi,c) \varPi} + \rho c \varPi \end{array} $$

where *N*_*ξ*_ = *D*_*ξ*_∇*ξ* ⋅∇*ξ*, *N*_*c*_ = *D*_*c*_∇*c* ⋅∇*c* and *N*_*ξc*_ = *D*∇*ξ* ⋅∇*c* are the scalar dissipation rates. The first and second line of Eq. 8 are identical with the original result by Bray et al. [21], which was extended in the present work to include the spray source terms. Note that in the derivation of Eq. 8 it is assumed that *D*_*c*_ = *D*_*ψ*_ = *D*_*ξ*_. For the sake of brevity, the entire term in the second line of Eq. 8 is written as $\theta \rho \dot {\omega }_{c}^{*}$. The presence of an evaporation source in the *c*-equation was discussed by Domingo et al. [7]. Using the jump conditions for *Y*_*ψ*_ and *ξ*, the first term in the third line can be expressed as the product of the evaporation mass source *ρ*π and a function,

9 $$\begin{array}{@{}rcl@{}} \mathscr{C}(\xi,c) = \frac{1}{\partial Y_{\psi}/\partial c} \left[ \delta_{\psi}-Y_{\psi} - (1-\xi) \frac{\partial Y_{\psi}}{\partial\xi} \right] \end{array} $$

which can be evaluated for any *ξ* and *c*, since *Y*_*ψ*_(*ξ*, *c*) is known from Eq. 7. In this expression *δ*_*ψ*_ is the mass fraction of species *ψ* in the liquid phase, which is equals 1 if *c*_*ψ*_ was based on the fuel and 0 otherwise. By grouping the terms in the Eq. 8, the c-equation finally appears in a a very similar form compared to Eq. 3 with one term representing the apparent reaction rate and two separate source terms due to evaporation.

The DCMC equation is the transport equation of the doubly conditional moments *Q*_*α*_ ≡〈*Y*_*α*_(**x**, *t*)|*ξ*(**x**, *t*) = *η*, *c*(**x**, *t*) = *ζ*〉. It is derived using the joint-pdf method assuming moderately high Reynolds number and invoking the primary closure hypothesis for the diffusive fluxes in conditional space [10]. This leads to,

10 $$\begin{array}{@{}rcl@{}} \frac{\partial Q_{\alpha}}{\partial t} + \langle \mathbf{u} | \eta, \zeta \rangle \cdot \nabla Q_{\alpha} &=& - \frac{1}{\bar{\theta}\bar{\rho}\widetilde{p}} \text{div} \left( \bar{\theta}\bar{\rho}\widetilde{p} \langle \mathbf{u}^{\prime\prime} Y_{\alpha}^{\prime\prime} | \eta, \zeta \rangle \right) \\ &&+ \frac{\text{Le}_{\xi}}{\text{Le}_{\alpha}} \langle N_{\xi}|\eta,\zeta\rangle \frac{\partial^{2} Q_{\alpha}}{\partial \eta^{2}} + \frac{\text{Le}_{c}}{\text{Le}_{\alpha}} \langle N_{c}|\eta,\zeta\rangle \frac{\partial^{2} Q_{\alpha}}{\partial \zeta^{2}} \\ &&+\left( \frac{\text{Le}_{\xi}}{\text{Le}_{\alpha}}+\frac{\text{Le}_{c}}{\text{Le}_{\alpha}}\right) \langle N_{\xi c}|\eta,\zeta\rangle \frac{\partial^{2} Q_{\alpha}}{\partial \eta \partial \zeta} \\ && + \langle \dot{\omega}_{\alpha} | \eta, \zeta \rangle - \langle \dot{\omega}_{c}^{*} | \eta, \zeta \rangle \frac{\partial Q_{\alpha}}{\partial \zeta} \\ &&+ \left( \delta_{\alpha} - Q_{\alpha} \right) \frac{\langle \varPi | \eta,\zeta \rangle}{\bar{\theta}} - \left[ \left( 1 - \eta \right) \frac{\partial Q_{\alpha}}{\partial \eta} + \mathscr{C}(\eta,\zeta) \frac{\partial Q_{\alpha}}{\partial \zeta} \right] \frac{\langle \varPi | \eta,\zeta \rangle}{\bar{\theta}} \\ &&- \frac{1}{\bar{\theta}\bar{\rho} \widetilde{p}} \frac{\partial \ \bar{\rho}\widetilde{p} (1-\eta) \langle Y_{\alpha}^{\prime\prime} \varPi^{\prime\prime} |\eta,\zeta\rangle}{\partial\eta} - \frac{1}{\bar{\theta}\bar{\rho} \widetilde{p}} \frac{\partial \ \bar{\rho}\widetilde{p} \mathscr{C}(\eta,\zeta) \langle Y_{\alpha}^{\prime\prime} \varPi^{\prime\prime} |\eta,\zeta\rangle}{\partial\zeta} \\ &&- \frac{1}{\bar{\theta}\bar{\rho} \widetilde{p}} \frac{\partial \ \bar{\theta}\bar{\rho}\widetilde{p} \langle Y_{\alpha}^{\prime\prime} \dot{\omega}_{c}^{*\prime\prime} |\eta,\zeta\rangle}{\partial\zeta} + \mathcal{D}_{Q \alpha} \end{array} $$

where 〈⋅|*η*, *ζ*〉 denotes density-weighted conditional averaging and $\widetilde {p}$ is the density-weighted pdf, defined as $\bar {\rho }\ \widetilde {p}(\eta )=\langle \rho |\eta ,\zeta \rangle p$. Moreover, $\bar {\theta }$ is the volume fraction of the gaseous phase, which is ≈ 1 in a dilute spray, and *δ*_*α*_ signifies the species mass fraction in the liquid phase, which is 1 for the liquid fuel species and 0 otherwise.

A similar transport equation can be derived for the conditional enthalpy *Q*_*h*_. This equation is presented in the Appendix and for completeness, the terms of molecular transport in the *Q*_*h*_-equation are also shown. The analogue term is neglected in Eq. 10 due to the high Reynolds number assumption.

In Eq. 10, $\dot {\omega }_{c}^{*}$ represents the apparent reaction progress variable source term in a flow with non-uniform mixture fraction. Since *Y*_*ψ*_ = *Y*_*ψ*_(*ξ*, *c*), according to the definition in Eq. 7, $Y_{\psi }^{\prime \prime }= 0$. Thus, the conditional apparent reaction rate is given as,

11 $$\begin{array}{@{}rcl@{}} \langle \dot{\omega}_{c}^{*}|\eta,\zeta\rangle = \frac{1}{\partial Q_{\psi}/\partial\zeta} \left[ \langle \dot{\omega}_{\psi}|\eta,\zeta\rangle + \langle N_{\xi}|\eta,\zeta\rangle \frac{\partial^{2} Q_{\psi}}{\partial\eta^{2}} + 2 \langle N_{\xi c}|\eta,\zeta\rangle \frac{\partial^{2} Q_{\psi}}{\partial \eta \partial \zeta} \right] \end{array} $$

Lewis number effects have been argued to be important for CMC of premixed flames [22]. Replacing the usual unity Lewis number assumption with the assumption of constant Le_*α*_ for each species, adds a factor to the scalar dissipation rate terms in Eq. 10 and also adds a differential diffusion term,

12 $$\begin{array}{@{}rcl@{}} \mathcal{D}_{Q \alpha}&= & \frac{1}{\bar{\theta}\bar{\rho}\widetilde{p}} \frac{\partial \ \bar{\theta}\bar{\rho}\widetilde{p} \langle (D_{\alpha} - D_{\xi}) \nabla \xi \cdot \nabla \xi |\eta, \zeta \rangle }{\partial \eta} \frac{\partial Q_{\alpha}}{\partial \eta} \\ && + \frac{1}{\bar{\theta}\bar{\rho}\widetilde{p}} \frac{\partial \ \bar{\theta}\bar{\rho}\widetilde{p} \langle (D_{\alpha} - D_{\xi}) \nabla \xi \cdot \nabla c | \eta, \zeta \rangle }{\partial \zeta} \frac{\partial Q_{\alpha}}{\partial \eta}\\ && + \frac{1}{\bar{\theta}\bar{\rho}\widetilde{p}} \frac{\partial \ \bar{\theta}\bar{\rho}\widetilde{p} \langle (D_{\alpha} - D_{c}) \nabla \xi \cdot \nabla c |\eta, \zeta \rangle }{\partial \eta} \frac{\partial Q_{\alpha}}{\partial \zeta}\\ && + \frac{1}{\bar{\theta}\bar{\rho}\widetilde{p}} \frac{\partial \ \bar{\theta}\bar{\rho}\widetilde{p} \langle (D_{\alpha} - D_{c}) \nabla c \cdot \nabla c |\eta, \zeta \rangle }{\partial \zeta} \frac{\partial Q_{\alpha}}{\partial \zeta} \end{array} $$

whose influence on major species might be small however [22]. We recall that in the derivation of the c-equation (8), it was assumed that Le_*c*_ =Le_*ξ*_ =Le_*ψ*_. This constraint can only be approximately valid in the general case of non-unity Lewis number and limits the choice of progress variable to major species with a Lewis number similar to Le_*ξ*_. If this limitation is respected and Le_*ψ*_ is sufficiently close to Le_*ξ*_, the error due this assumption will be small compared to the effect of non-unity Le_*α*_ which for minor species can be substantially different from Le_*ξ*_.

Considering the effects of liquid fuel evaporation adds several terms to the DCMC equation (10), including doubly conditional terms equivalent to the terms first derived by Mortensen and Bilger [12]. Additional new terms appear to account for the effects of evaporation on the reaction progress variable. These terms contain the function $\mathscr {C}(\eta ,\zeta )$, which is given by Eq. 9.

In the present derivation, the primary closure hypothesis is specifically applied to fluxes of the reactive scalar in conditional space only. This is in line with the original derivation [23], where it is clearly specified that closure is achieved by assuming a “first-order diffusion relation” for these fluxes. Source terms from evaporation or the reaction progress variable are not treated through this approximation, which leads to the appearance of terms containing the conditional correlations 〈*Y* *α*″π^″^|*η*, *ζ*〉 and $\langle Y_{\alpha }^{\prime \prime }\dot {\omega }_{c}^{*\prime \prime }|\eta ,\zeta \rangle $. In this way, Mortensen and Bilger [12] derived the conditional correlation term of mass fraction and evaporation rate using the joint-pdf method, whilst it seems that this term does not appear when the decomposition method is used. Indeed, the conditional correlation term of mass fraction and reaction progress variable source does not occur in the CMC equation for premixed flames derived by Mantel and Bilger [24] using the decomposition method and it has not been addressed in more recent work [22, 25] either. The effect of the conditional correlation term of the evaporation rate has not been studied yet and it has been neglected so far [13, 26].

### Closure for the DCMC equation

Equation 10 represents the unclosed DCMC equation in its general form, whose derivation only requires very light modelling assumptions, viz. the primary closure hypothesis, moderate to high Reynolds number and constant Lewis numbers. Whilst the accuracy of first order closure for the doubly conditional reaction source term has been demonstrated [16], there is, in general, very little experience with the sub-models for DCMC. In some cases, however, it might be possible to generalise models used in conventional CMC or to adapt them from other combustion models that use a similar parametrisation, such as for instance mixture fraction-progress variable flamelet models.

In order to apply the DCMC model to a preliminary test case, in the present work the modelling choices detailed below are made. The selection of sub-models was partly driven by their extremely limited availability. In this sense, the set of models proposed is a first suggestion and more work will be necessary in the future to improve and validate sub-models for DCMC.

Unity Lewis number is assumed for simplicity in this first application of the model. For the transport in physical space the well-tested sub-models for conventional CMC can be easily adopted. The diffusion approximation is used for 〈**u** ^″^ *Y* *α*″|*η*, *ζ*〉 [10] and the conditional velocity is modelled as $\langle \mathbf {u}|\eta ,\zeta \rangle =\widetilde {\mathbf {u}}$, since gradients of $\widetilde {\xi }$ are small. For the Favre pdfs of the conditioning variables, *β*-pdfs are presumed and, assuming statistical independence, the joint-pdf is computed as $\widetilde {p}(\eta ,\zeta )=p_{\beta }(\eta ; \widetilde {\xi }, \widetilde {\xi ^{\prime 2}}) p_{\beta }(\zeta ; \widetilde {c}, \widetilde {c^{\prime 2}})$. This assumption is made for simplicity since accurate modelling of the joint-pdf in the present three-stream mixing problem plus droplet evaporation would be very complex and is not attempted here. The choice of sub-models for the doubly conditional scalar dissipation rates is very limited. In the present work, we follow the suggestions by Nguyen et al. [27]. For the scalar dissipation rate of the mixture fraction they assumed that *N*_*ξ*_ is primarily imposed by the mixing with little dependence on chemistry and, thus, 〈*N*_*ξ*_|*η*, *ζ*〉≈〈*N*_*ξ*_|*η*〉, which is modelled as a bell curve given by the Amplitude Mapping Closure (AMC) model [28]. Again following Nguyen et al. [27], 〈*N*_*c*_|*η*, *ζ*〉 is modelled as the product of two bell curves, *b*(*η*) centred on the stoichiometric mixture fraction *η* = *ξ*_st_ and *G*(*ζ*), centred on *ζ* = 0.5; for *η* ≥ 2*ξ*_st_ it is zero. This model is a simple approximation of *N*_*c*_-values from premixed flamelet tabulation. The conditional cross-scalar dissipation rate is not considered, in line with the assumption of statistically independent conditioning variables in the modelling of the pdf. A dilute spray is assumed, i.e. $\bar {\theta }\approx 1$ but the doubly conditional evaporation source terms are not included in the DCMC equation. Finally radiation and wall heat losses are neglect. Together with the unity Lewis number assumption and in the absence spray source terms, the transport equation of the conditionally averaged enthalpy *Q*_*h*_ becomes trivial and does not need to be calculated. The conditional temperature *Q*_*T*_ is then calculated from *Q*_*h*_ and the conditional species mass fractions *Q*_*α*_.

### Flow field solver

The DCMC combustion model is coupled to the flow field solver. An Euler-Lagrangian approach is followed, where the gaseous phase is computed through an unsteady Reynolds-averaged Navier-Stokes (RANS) simulation with the standard *k*-*ε* turbulence model and the parcels of liquid droplets are tracked as Lagrangian particles. The flow field solver integrates the momentum equation as well as the Favre-mean and variance equations of the conditioning variables, *ξ* and *c*. Note that (⋅)^′^ denotes the fluctuation around the Favre mean, i.e. $Y=\widetilde {Y}+Y^{\prime }$, to distinguish it from the conditional fluctuation in the DCMC equation. Here, the reaction progress variable is based on the mass fraction of carbon dioxide, $Y_{\psi }=Y_{\text {CO}_{2}}$.

13 $$\begin{array}{@{}rcl@{}} \frac{\partial \bar{\rho} \widetilde{\xi}}{\partial t} + \text{div}(\bar{\rho} \widetilde{\xi} \widetilde{\mathbf{u}}) = \text{div}\left( \bar{\rho} (D_{T} + D) \nabla\widetilde{\xi} \right) + \bar{\rho}\widetilde{ \varPi} \end{array} $$

14 $$\begin{array}{@{}rcl@{}} \frac{\partial \bar{\rho} \widetilde{\xi^{\prime 2}}}{\partial t} + \text{div}(\bar{\rho} \widetilde{\xi^{\prime 2}} \widetilde{\mathbf{u}}) &=& \text{div}\left( \bar{\rho} (D_{T} + D) \nabla\widetilde{\xi^{\prime 2}} \right) - 2\bar{\rho}\ \frac{\widetilde{\varepsilon}}{\widetilde{k}}\ \widetilde{\xi^{\prime 2}} + 2\bar{\rho} D_{T} \nabla\widetilde{\xi}\cdot\nabla\widetilde{\xi} \\ &&+ 2\bar{\rho}(\widetilde{\xi\varPi}-\widetilde{\xi}\widetilde{\varPi}) - \bar{\rho}(\widetilde{\xi^{2}\varPi}-{\widetilde{\xi}}^{2}\widetilde{\varPi}) \end{array} $$

15 $$\begin{array}{@{}rcl@{}} \frac{\partial \bar{\rho} \widetilde{c}}{\partial t} + \text{div}(\bar{\rho} \widetilde{c} \widetilde{\mathbf{u}}) = \text{div}\left( \bar{\rho} (D_{T} + D) \nabla\widetilde{c} \right) + \bar{\rho}\widetilde{\dot{\omega}_{c}^{*}} + \bar{\rho}\widetilde{\mathscr{C}\varPi} + \bar{\rho}\widetilde{c\varPi} \end{array} $$

16 $$\begin{array}{@{}rcl@{}} \frac{\partial \bar{\rho} \widetilde{c^{\prime 2}}}{\partial t} + \text{div}(\bar{\rho} \widetilde{c^{\prime 2}} \widetilde{\mathbf{u}}) &=& \text{div}\left( \bar{\rho} (D_{T} + D) \nabla\widetilde{c^{\prime 2}} \right) - 2\bar{\rho} \widetilde{\varepsilon}_{c} + 2\bar{\rho} D_{T} \nabla\widetilde{c}\cdot\nabla\widetilde{c} \\ &&+ 2 \bar{\rho}\widetilde{c^{\prime}\dot{\omega}_{c}^{*\prime}} + 2 \bar{\rho} (\widetilde{c \mathscr{C}\varPi} - \widetilde{c}\widetilde{\mathscr{C}\varPi} ) + \bar{\rho} (\widetilde{c^{2}\varPi} -2 \widetilde{c}\widetilde{c\varPi} +\widetilde{c}^{2}\widetilde{\varPi} ) \end{array} $$

The eddy diffusivity is calculated as $D_{T}= \mu _{T}/(\bar {\rho }\text {Sc}_{T})$, with a constant turbulent Schmidt number Sc_*T*_ = 0.7; for the molecular diffusivity $D= \mu /(\bar {\rho }\text {Sc})$, with Sc = 0.7. In the case of the mixture fraction, the scalar dissipation rate is modelled as for a passive scalar, $(\widetilde {\varepsilon }/\widetilde {k})\ \widetilde {\xi ^{\prime 2}}$ [29]. The scalar dissipation rate of *c* is closed using the model by Kolla et al. [30],

17 $$\begin{array}{@{}rcl@{}} \widetilde{\varepsilon}_{c} = \frac{1}{\beta^{\prime}} \left[ \left( 2K_{c}^{*} - \tau(\widetilde{\xi}) C_{4} \right) \frac{{S_{L}^{0}}(\widetilde{\xi})} {{\delta_{L}^{0}}(\widetilde{\xi})} + C_{3} \frac{\widetilde{\varepsilon}}{\widetilde{k}} \right] \widetilde{c^{\prime 2}} \end{array} $$

where the model coefficients are set as described by Kolla and Swaminathan [31], notably *β* ^′^ = 6.7 and $K_{c}^{*}= 0.85\tau $. To account for non-uniform mixture *τ*, ${S_{L}^{0}}$ and ${\delta _{L}^{0}}$ are evaluated at the local mean mixture fraction $\widetilde {\xi }$ [32]. For this purpose, the laminar flame speed and the thermal laminar flame thickness are pre-computed using the commercial software Cosilab and tabulated as functions of mixture fraction, ${S_{L}^{0}}(\xi )$ and ${\delta _{L}^{0}}(\xi )$ respectively; $\tau (\widetilde {\xi })$ is calculated based on the conditional temperature *Q*_*T*_(*η*, *ζ*) with $\eta =\widetilde {\xi }$ and the Karlovitz number is $\text {Ka}=[{\delta _{L}^{0}}(\widetilde {\xi })/{S_{L}^{0}}(\widetilde {\xi })]/\sqrt {\nu /\widetilde {\varepsilon }}$.

The reaction source term in the mean and variance equation of the progress variable, $\widetilde {\dot {\omega }_{c}^{*}}$ and $\widetilde {c^{\prime }\dot {\omega }_{c}^{*\prime }}$, are calculated by integrating $\langle \dot {\omega }_{c}^{*}|\eta ,\zeta \rangle $ (11) with the pdf in the conditional space.

18 $$\begin{array}{@{}rcl@{}} \widetilde{\dot{\omega}_{c}^{*}} = {{\int}_{0}^{1}} {{\int}_{0}^{1}} \langle \dot{\omega}_{c}^{*}|\eta,\zeta \rangle \ \widetilde{p}(\eta,\zeta) \ \mathrm{d}\eta\ \mathrm{d}\zeta \end{array} $$

19 $$\begin{array}{@{}rcl@{}} \widetilde{c^{\prime}\dot{\omega}_{c}^{*\prime}} = {{\int}_{0}^{1}} {{\int}_{0}^{1}} \zeta\ \langle \dot{\omega}_{c}^{*}|\eta,\zeta \rangle \ \widetilde{p}(\eta,\zeta) \ \mathrm{d}\eta\ \mathrm{d}\zeta - \widetilde{c}\ \widetilde{\dot{\omega}_{c}^{*}} \end{array} $$

**Fig. 1:**
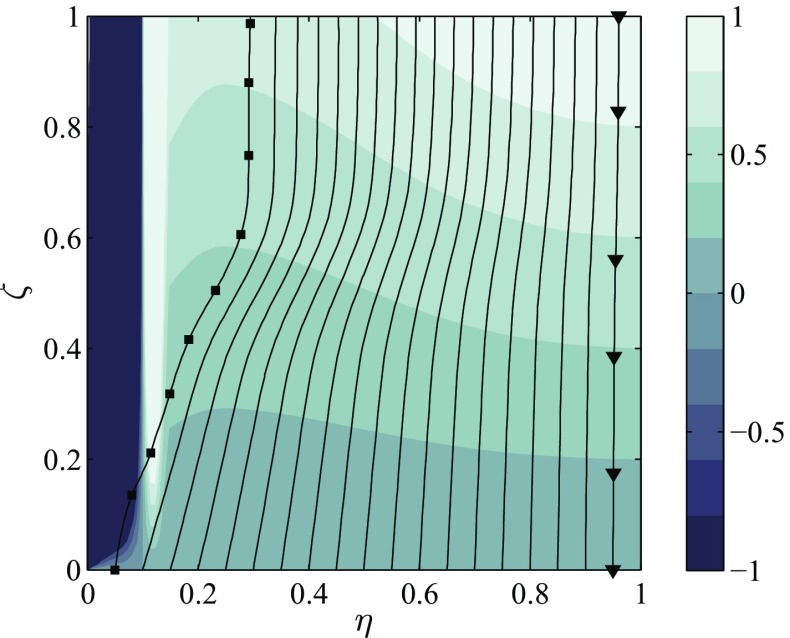
Closure model for Favre-averaged spray source terms. The contour plot represents $(\mathscr {C}(\eta ,\zeta ) + \zeta )$, which is overlaid with black iso-lines of fuel mass fraction *Y*_*F*_. The range of $(\mathscr {C}+\zeta )$ is cropped at − 1 and + 1

In this work an emphasis is put on the modelling of the spray combustion and we aim to provide closure for the complete set of evaporation terms that appear in the Favre-averaged transport equations of the conditioning variables. The mean evaporation source is computed by summing over the evaporated mass of all droplets in a CFD cell,

20 $$\begin{array}{@{}rcl@{}} \widetilde{\varPi} = \frac{1}{\bar{\rho} V} \sum\limits_{i}^{N_{d}} \dot{m}_{d,i} \end{array} $$

where *V* represents the cell volume. The Favre-averaged transport equations contain several other evaporation source terms, which require modelling. In particular, for the mixture fraction variance equation, Giusti and Mastorakos [33] pointed out that both spray source terms could have a significant effect in the inner flame region. In single-conditional CMC, the terms $\widetilde {\xi \varPi }$ and $\widetilde {\xi ^{2}\varPi }$ are modelled either by summing $\xi _{s,i}^{k} \dot {m}_{d,i}$ (*k* = 1 or 2) for all droplets in one cell [34] or, alternatively, by assuming that 〈π|*η*〉 has the shape of a *δ*-function at the average surface mixture fraction 〈*ξ*_*s*_〉 [35]. In both cases, it is assumed that *ξ*_*s*_ ≈ *Y*_*F*,sat_, calculated for the droplet temperature *T*_*d*_. This assumption is, however, only correct if the droplets evaporate upstream of the flame [13]. In general, $Y_{F,\text {sat}} \leq \xi _{s} \leq Y_{F,\text {Eq}}^{-1}(Y_{F,\text {sat}})$, where $Y_{F,\text {Eq}}^{-1}$ is the inverse function of the equilibrium fuel mass fraction *Y*_*F*,Eq_(*ξ*). This reflects the case of a droplet evaporating in burned gases where some reaction products are present besides the fuel vapour at the droplet surface. In principle, this effect can be accounted for by integrating the spray source term in doubly conditional space. For a generic spray source term of the type $\widetilde {\mathcal {F}\varPi }$, Eq. 21 is exact if $\langle \mathcal {F}^{\prime \prime }\varPi ^{\prime \prime }|\eta ,\zeta \rangle = 0$, notably for $\mathcal {F}=\xi $ or $\mathcal {F}=\mathscr {C}(\xi ,c)$ etc. Fig. 1 shows a contour plot of the spray source term of the $\widetilde {c}$-equation in conditional space, $(\mathscr {C}(\eta ,\zeta )+\zeta )$, which can be directly evaluated using the conditional moments from CMC. Considering the conditional evaporation term shows that two necessary conditions for a source term of the progress variable are fulfilled: first, droplet evaporation in unburned mixture does not impact the progress variable since $(\mathscr {C}(\eta ,\zeta )+\zeta )= 0$ for *ζ* = 0 and, second, $(\mathscr {C}(\eta ,\zeta )+\zeta )\leq 1$ for *ζ* = 1, which signifies *c* is automatically bounded at 1 with respect to this term.

In order to devise a model for 〈π|*η*, *ζ*〉 it is recognised that the droplet temperature computed by the evaporation model fixes the fuel mass fraction – not the mixture fraction – on the droplet surface. This means that the droplet surface corresponds to the isoline of *Q*_*F*_(*η*, *ζ*) = *Y*_*Fs*_ in conditional space (represented by black lines in Fig. 1). Since evaporation only happens on the droplet surface, 〈π|*η*, *ζ*〉 must be zero everywhere except on this isoline and $\widetilde {\mathcal {F}\varPi }$ can be calculated as a line integral. In Eq. 22, it was further assumed that π(*η*, *ζ*) is constant along this isoline. This is consistent with the evaporation model, where $\dot {m}_{d}$ is computed based on the droplet temperature and the temperature far from the droplet surface, thus ignoring the exact temperature distribution in the near field.

21 $$\begin{array}{@{}rcl@{}} \widetilde{\mathcal{F}\varPi} &\approx& {{\int}_{0}^{1}} {{\int}_{0}^{1}} \langle\mathcal{F}|\eta,\zeta\rangle\ \langle\varPi|\eta,\zeta\rangle\ \widetilde{p}(\eta,\zeta)\ \mathrm{d}\eta\ \mathrm{d}\zeta \end{array} $$

22 $$\begin{array}{@{}rcl@{}} &\approx& \frac{\widetilde{\varPi}}{{\int}_{C} \widetilde{p}(\eta,\zeta)\ \mathrm{d}s } {\int}_{C} \langle\mathcal{F}|\eta,\zeta\rangle\ \widetilde{p}(\eta,\zeta)\ \mathrm{d}s \end{array} $$

Note that the model of 〈π|*η*, *ζ*〉 presented above is only used to close the Favre-averaged evaporation source terms, but it is not used as a sub-model in the DCMC equation (see Section 2.2).

The evaporation of the Lagrangian droplets was computed according to the model by Abramzon and Sirignano [36] with Stefan flow correction and non-unity Lewis number in the film; for the liquid droplets, the approximation of infinite conductivity is made.

23 $$\begin{array}{@{}rcl@{}} \frac{\mathrm{d}m_{d}}{\mathrm{d}t} = -\dot{m}_{d} = - \varPi d_{d} \rho_{G} D_{G} \text{Sh}^{*} \ln(1+B_{M}) \end{array} $$

24 $$\begin{array}{@{}rcl@{}} \frac{\mathrm{d}T_{d}}{\mathrm{d}t} = - \frac{1}{m_{d} C_{pL}} \frac{\dot{m}_{d} C_{pV}}{B_{T}} (\widetilde{T}-T_{d}) + \frac{1}{m_{d} C_{pL}} \dot{m}_{d} L_{V} \end{array} $$

where *B*_*M*_ and *B*_*T*_ are the Spalding mass and heat transfer numbers respectively. Sh and Nu are calculated using the Frössling correlation. The quantities *ρ*_*G*_, *C*_*pG*_, *C*_*pV*_, *μ*_*G*_, *λ*_*G*_, *D*_*G*_ are evaluated at reference conditions for the gaseous boundary layer, computed according to the 1/3 rule for temperature and species; *ρ*_*L*_, *C*_*pL*_, latent heat *L*_*V*_ and the fuel saturation vapour pressure $p_{F}^{\text {sat}}$ are computed at the droplet temperature *T*_*d*_. The droplet experiences sphere drag and the impact of turbulence on the droplet motion is mimicked through stochastic dispersion for isotropic turbulence. No model for secondary break-up of droplets was used.

### Chemistry

A detailed chemical mechanism for ethanol combustion [37] with 57 species and 383 reversible reactions is used. The mechanism performs well in predicting ignition delays and laminar flame speeds at ambient pressure when compared to experimental data. It has been successfully used in a CMC simulation by Giusti and Mastorakos [33].

### Numerical set-up

The computational fluid dynamics toolbox OpenFOAM-2.3.0 is used to solve for the Favre-averaged flow field variables, $\widetilde {\mathbf {u}}$, $\widetilde {\xi }$, $\widetilde {\xi ^{\prime 2}}$, $\widetilde {c}$ and $\widetilde {c^{\prime 2}}$ and to track the Lagrangian droplets. The coupling of the flow field solver and the DCMC model is effectuated as shown in a schematic in Ref. [38]. In addition to $\bar {\rho }$ and $\widetilde {T}$, the DCMC model also returns $\widetilde {Y_{F}}$, $\widetilde {\dot {\omega }_{c}^{*}}$, $\widetilde {c^{\prime }\dot {\omega }_{c}^{* \prime }}$ and the Favre-averaged evaporation terms (22) to the flow field solver. The motion and evaporation of the Lagrangian droplets are solved at the at beginning of each time step.

Applying the closure models and simplifying assumptions presented in Section 2.2, the DCMC equation (10) reduces to,

25 $$\begin{array}{@{}rcl@{}} \frac{\partial Q_{\alpha}}{\partial t} +\text{div}\left( Q_{\alpha}\widetilde{\mathbf{u}}\right) &=& Q_{\alpha}\ \text{div}\ \widetilde{\mathbf{u}} + \frac{\mu_{T}}{\bar{\rho} Sc_{T}} \nabla Q_{\alpha} \\ &&+\langle N_{\xi}|\eta,\zeta\rangle\frac{\partial^{2} Q_{\alpha}}{\partial\eta^{2}} + \langle N_{c}|\eta,\zeta\rangle\frac{\partial^{2} Q_{\alpha}}{\partial\zeta^{2}} \\ &&-\frac{\partial Q_{\alpha}}{\partial\zeta} \frac{1}{\partial Q_{\psi}/\partial\zeta} \left( \langle N_{\xi}|\eta,\zeta\rangle\frac{\partial^{2} Q_{\psi}}{\partial\eta^{2}} + \langle N_{c}|\eta,\zeta\rangle\frac{\partial^{2} Q_{\psi}}{\partial\zeta^{2}} \right)\\ &&+\langle\dot{\omega}_{\alpha}|\eta,\zeta\rangle -\frac{\partial Q_{\alpha}}{\partial\zeta} \frac{1}{\partial Q_{\psi}/\partial\zeta} \langle\dot{\omega}_{\psi}|\eta,\zeta\rangle \end{array} $$

Eq. 25 is integrated with a finite volume method whilst employing an operator splitting procedure. The fractional step approach is well established in CMC modelling and the significance of operator splitting errors in spray flames was investigated by Wright et al. [39]. The operator splitting approach allows for higher computational efficiency, since the stiff chemistry term is decoupled from the non-stiff convective terms. First, the transport in physical space by advection and turbulent diffusion, as well as the dilatation term (terms 2, 3 and 4 in the first line of Eq. 25 respectively) are computed. An upwind scheme is used for the advection term. Second, transport in conditional space (lines 2 and 3 in Eq. 25) is integrated for every DCMC cell. Finally, the chemical reaction source is solved independently for every (*η*, *ζ*)-node in every DCMC cell. For the second and the third sub-step the solver VODPK is used. This operator splitting procedure automatically assures that *Q*_*ψ*_ conserves its linear dependence on *ζ* according to the definition of the progress variable.

A 1D DCMC grid was used to discretise the physical domain with 35 DCMC cells along the burner axis for 0 < *z*/*d* < 5. The transfer of data from the fine CFD mesh to the coarse DCMC grid is achieved by integrating the conditional scalar dissipation rates over the volume of the DCMC cell [40].

The conditional space, $D=\{(\eta ,\zeta )\in \mathbb {R}^{2}: 0\le \eta \le 1, 0\le \zeta \le 1\}$, is discretised with 51 *η*-nodes, clustered around the stoichiometric mixture fraction *η* = *ξ*_st_ (for the reaction of ethanol with air *ξ*_st_ ≈ 0.1006) and 41 *ζ*-nodes, which are more closely spaced at *ζ* = 1. The derivatives in conditional space are discretised with second-order finite differences apart from *∂*/*∂* *ζ*, which is computed with an upwind scheme. Dirichlet boundary conditions are set on all four sides of the conditional domain; mixing line and equilibrium condition for *ζ* = 0 and 1 respectively, air at *η* = 0 and fuel vapour at the boiling point at *η* = 1. The equilibrium condition was approximated by solving single-conditioned, non-premixed “0D-CMC” equation, similar to the non-premixed flamelet equation, with a very low scalar dissipation rate *N*_*ξ*,max_ = 1 1/s, compared to the critical scalar dissipation rate of approximately 367 1/s [41]. The initial condition for the doubly conditional moments in the DCMC cells is computed by solving the 0D-DCMC equation, that is to say the DCMC equation (25) with prescribed, fixed scalar dissipation rates and for a spatially homogeneous case, i.e. without the terms that represent transport in physical space, until steady state is reached. The solutions of this equation are similar to the results presented by Nguyen et al. [27]. As DCMC initial condition a very weakly strained solution of the 0D-DCMC equation was used; notably, *N*_*ξ*,max_ = 1 1/s and *N*_*c*,max_ = 200 1/s. The set of conditional moments used as inlet boundary condition is identical with the initial condition. Hence the conditional moments in the DCMC cell located at *z*/*d* = 0, shown later in Figs. 4 and 5, are representative of the DCMC initial condition.

### Experimental test case

The DCMC model is applied to a piloted ethanol spray flame recently studied experimentally by Kariuki and Mastorakos [17]. The presence of droplet evaporation and, thus, the existence of mixture inhomogeneities, combined with substantial pre-vaporisation and premixing makes this flame a suitable candidate to test the present DCMC model.

In the experimental set-up, the ethanol spray is injected into the main flow of air ≈ 38 cm upstream of the nozzle with a diameter *d* = 42 mm. The flame is stabilised at the end of the nozzle through an annular pilot of diameter ≈ 51 mm and width ≈ 6 mm. The pilot is a open premixed methane-air flame with an approximately stoichiometric fuel-air ratio and a cold volume flow rate of 33.5 L/min. In the present work, we consider two reacting cases with overall equivalence ratios (liquid fuel to main air flow) *ϕ* = 0.62 and 0.82 (corresponds to *ξ* ≈ 0.065 and 0.084 respectively) and a non-reacting case. In all three cases the air-flow rate is 235 L/min and the liquid fuel mass injected in the cold case is the same as in the richer flame. Hence, the flames have the character of a pilot-stabilised turbulent premixed jet flames with a bulk velocity *U*_*b*_ ≈ 3.04 m/s, calculated using the area of the nozzle.

The three-dimensional computational domain stretches − 1 < *z*/*d* < 19 along the burner axis, where *z* = 0 is the position of the nozzle outlet and the pilot and the main flow inlet is retracted by one nozzle diameter; the diameter of the entire domain is 33*d*. Turbulence levels are set according to Laser Doppler Anemometry (LDA) measurements to *u* ^′^/*U*_*b*_ ≈ 0.18 and estimating *L*_*T*_ ≈ *d*/3. Since the degree of pre-vaporisation of the ethanol spray was not measured in the experiment, the mixture fraction at the inlet had to be estimated. According to a separate RANS simulation of the upstream part of the burner corresponding to the richer flame and the cold case, the inlet boundary condition for the mixture fraction was set to $\widetilde {\xi }= 0.04$ and $\widetilde {\xi ^{\prime 2}}= 0$. Even though the injected amount of liquid fuel is smaller in the leaner case, the same boundary condition is also used for the simulation of the flame with *ϕ* = 0.62. This will allow us to explore the sensitivity of the simulation results to the level of pre-vaporisation and premixing in the upstream region of a spray flame; in the rich flame approximately half the fuel is pre-vaporised compared to two thirds in the leaner flame. For the main flow, the inlet boundary condition for the reaction progress variable upstream of the nozzle is set to $\widetilde {c}= 0$. For the purpose of the present paper whose aim is to discuss the model and its application, the uncertainty related to the inlet boundary condition is considered satisfactory.

The pilot is an open flame, which is not retracted relative to the main flow. Hence, dilatation in the pilot flame does not lead to a significant increase of the mean axial velocity but to an increase in width of the hot pilot stream as it is expected for a usual triangular flame. Following a single mixture fraction based approach in the simulations, the pilot is modelled as a premixed stoichiometric ethanol-air flame. The annular pilot flame itself is not resolved but instead modelled as a uniform inlet boundary condition with a laminar inflow of burnt gases, i.e. $\widetilde {\xi }=\xi _{\text {st}}$, $\widetilde {\xi ^{\prime 2}}= 0$ and $\widetilde {c}= 1$, with a mass flow rate corresponding to the experimental configuration. In the experimental rig the annulus that stabilises the pilot is very narrow and the pilot flow is broadened by the dilatation across the flame. If the width of the original annulus was used for the uniform inlet of hot gas, whilst the pilot mass flow rate was kept equal to its value in the experiments, the axial velocity of the pilot flow would be greatly over-estimated. Instead, the surface area of the pilot inlet is increased to assure a realistic axial velocity for the pilot flow. For this purpose, the surface of the pilot inlet is three times the surface area of the annulus in the rig.

A small, laminar co-flow of 0.1 m/s is set around the burner nozzle, where $\widetilde {\xi }= 0$ and $\widetilde {c}= 1$. Walls are considered adiabatic.

## Results and Discussion

**Fig. 2:**
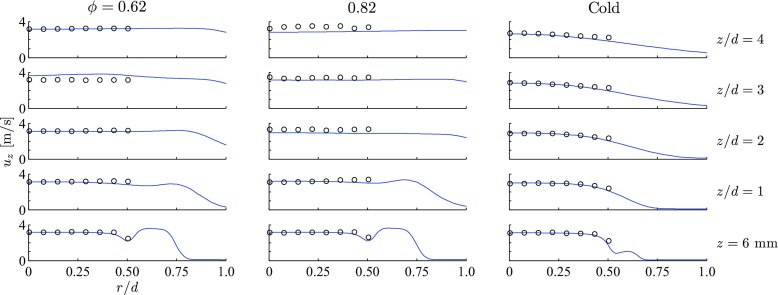
Radial profiles of mean axial velocity, 〈*u*_*z*_〉, $\widetilde {u}_{z}$ for the cold flow and the flames; the mean gas phase velocity from the RANS simulation (line –) is compared to PDA measurements of droplet velocity (symbols ∘)

Figure 2 shows the radial profiles of the mean axial velocity of the gas phase for cold flow and the two flames considered here. The results are compared to Phase Doppler Anemometry (PDA) measurements of the mean axial velocity of the droplets in the range 0 ≤ *r*/*d* ≤ 0.5, not including the pilot stream, by Kariuki and Mastorakos [17]. In the cold case, the effect of the pilot stream is small and the flow spreads like a turbulent jet with a radial profile similar to a Gaussian bell curve. In contrast, for both flames studied, the axial mean velocity is almost constant for *r*/*d* < 0.5. This feature is well reproduced by the simulation.

**Fig. 3:**
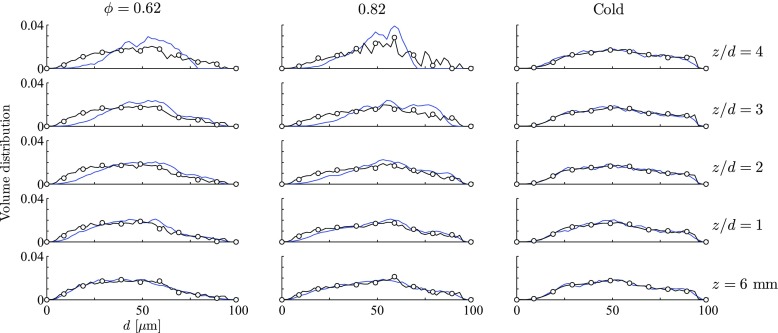
Droplet volume distributions; the mean gas phase velocity from the RANS simulation (line –) is compared to PDA measurements of droplet velocity (line with symbols ∘). The distributions are normalised to integrate to one

Next the droplet size information from the PDA measurements [17] is compared to simulation results. Figure 3 shows volume distributions of droplet size. The spray is injected far upstream of the nozzle and experimental measurements show that the droplet distributions are very similar at different radial positions in the core of the main flow, even for reacting cases. Thus, droplet distributions are only shown for different axial positions, considering the experimental data from all measurement points with *r* < 0.75 ⋅ (*d*/2). For the simulations, the droplet volume distributions at the inlet were set equal to the experimental ones from the axial position *z* = 6 mm. In general, a reasonable agreement between the experimental volume distributions and the simulation results is achieved. In both flames, the droplet volume distribution flattens in the range of small droplets (*d* < 30 *μ* m) whilst it increases for larger droplets (40 *μ* m < *d* < 70 *μ* m). This can be explained by (i) a shorter heating-up period and thus quicker evaporation of smaller droplets and (ii) a faster decrease in diameter for smaller droplets when their temperature is nearly constant and *d* ^2^ is known to decrease approximately linearly in time. The phenomenon is slightly over-predicted in the simulations but can also be observed in the PDA data, in particular, for the richer flame.

**Fig. 4:**
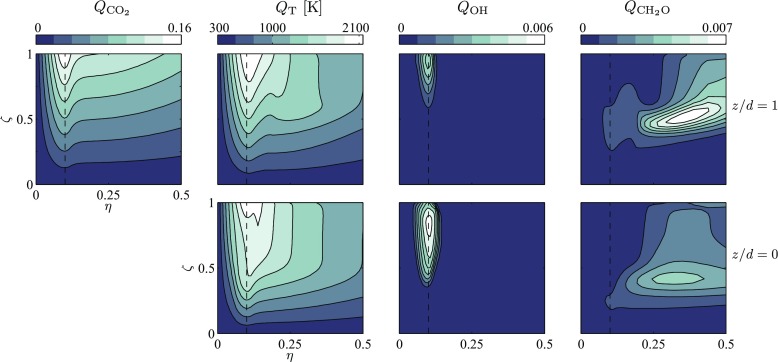
Conditional moments of $Y_{\text {CO}_{2}}$, *T*, *Y*_OH_ and $Y_{\text {CH}_{2}\mathrm {O}}$ in two different DCMC cells for the *ϕ* = 0.82 flame. The DCMC cells are located at *z*/*d* = 0 and *z*/*d* = 1. The dashed line marks the stoichiometric mixture fraction *ξ*_st_ ≈ 0.1005. Note that only the range 0 ≤ *η* ≤ 0.5 is shown, but the DCMC equation is solved for all 0 ≤ *η* ≤ 1

In Fig. 4 the conditional moments of CO_2_, OH, CH_2_O and temperature are shown for two different DCMC cells of the richer flame. Due to the definition of the progress variable based on the mass fraction of CO_2_, the conditional moment $Q_{\text {CO}_{2}}$ is fixed for all DCMC cells equal to its shape defined by Eq. 7. The DCMC cell at *z*/*d* = 0 is located at the exit of the nozzle very close to the inlet and the scalar dissipation rates upstream of the flame are low. Hence, the structure of a weakly strained flame is mostly influenced by the advective term of the DCMC equation. The conditional moments shown here are almost identical with the DCMC inlet boundary condition and the DCMC initial condition. For *η* = *ξ*_st_ the temperature rises quickly for 0 ≤ *ζ* < 0.5 and then flattens out for higher *ζ*, slowly approaching the temperature at equilibrium condition. In contrast, the DCMC cell at *z*/*d* = 1 contains the flame and thus experiences increased scalar dissipation rates. In particular, a high $\widetilde {N_{c}}$ diffuses reactants in conditional space. Consequently, *Q*_*T*_ rises almost linearly from *ζ* = 0 to 1 at *η* = *ξ*_st_. In the region further downstream of the flame $\widetilde {N_{c}}= 0$ and $\widetilde {N_{\xi }}$ decays. As a result, the conditional moments experience less straining due to the scalar dissipation rates and slowly relax back to the structure of the weakly strained flame.

**Fig. 5:**
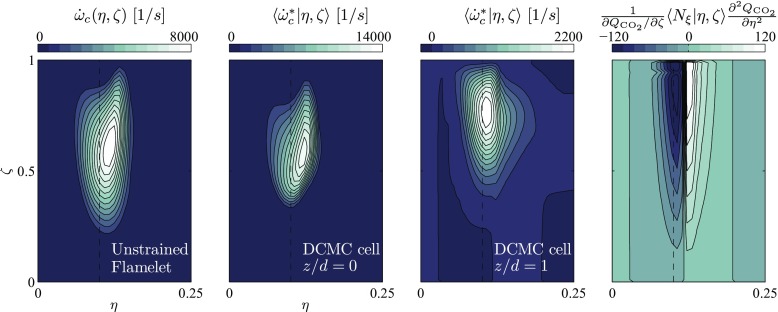
Conditional reaction progress variable source term. From left to right: unstrained premixed flamelet $\dot {\omega }_{c}$, $\langle \dot {\omega }_{c}^{*}|\eta ,\zeta \rangle $ in a DCMC cell at *z*/*d* = 0, and another DCMC cell at *z*/*d* = 1, as well as the non-premixed contribution to $\langle \dot {\omega }_{c}^{*}|\eta ,\zeta \rangle $ for the DCMC cell at *z*/*d* = 1. Simulation results for the case with *ϕ* = 0.82

The conditional apparent reaction rates computed in the two DCMC cells previously discussed are shown in Fig. 5. They are compared to $\dot {\omega }_{c}(\eta ,\zeta )$ from unstrained premixed flamelets, which were computed using the commercial software Cosilab. In the weakly strained case at *z*/*d* = 0 the conditional reaction rate reaches significantly higher values than for the DCMC cell located at *z*/*d* = 1. This shows how the conditional reaction rate adjusts as the scalar dissipation rates increase. Moreover, in the strained flame $\langle \dot {\omega }_{c}^{*}|\eta ,\zeta \rangle $ also takes negative values. This is due to the contribution of the non-premixed term, which is also shown in Fig. 5.

**Fig. 6:**
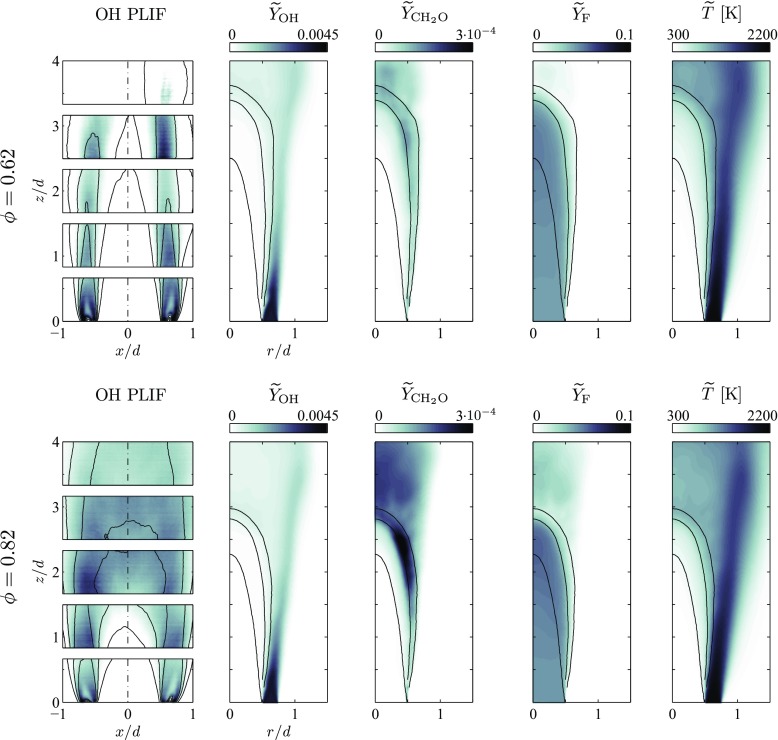
Ensemble-averaged OH-PLIF measurements overlaid with experimentally computed mean progress variable 〈*c*〉 isolines [17], compared to $\widetilde {Y}_{\text {OH}}$ and $\widetilde {c}$-isolines from the simulations, for both flame cases. The isolines are for the values 0.1, 0.5 and 0.9. Fields of $\widetilde {Y}_{\text {CH}_{2}\mathrm {O}}$, $\widetilde {Y}_{\mathrm {F}}$, $\widetilde {T}$ from the simulation are also shown. Note the difference in the range of the *x*-axis and *r*-axis

Next the shape of the flames is assessed. Figure 6 shows sample averaged OH planar laser-induced fluorescence (OH-PLIF) images by Kariuki and Mastorakos [17], in comparison to $\widetilde {Y}_{\text {OH}}$ from the present simulations. Comparisons are qualitative and no scale is shown for the mean OH PLIF intensity. On top of the simulation data $\widetilde {c}$-isolines are plotted to emphasis the position and width of the flame brush. They can be directly compared to isolines of ensemble-averaged progress variable based on experimental data, even at a quantitative level. For this purpose, the instantaneous field of progress variable was determined experimentally by tracking the flame front through a thresholding procedure applied to the OH PLIF images [17]. For the richer flame with an overall equivalence ratio of *ϕ* = 0.82 (Fig. 6, bottom), the mean flame brush is relatively broad, occupying the range 1 < *z*/*d* < 2.5 along the burner axis, where the mean progress variable increases from 0.1 to 0.9. The simulation predicts a slightly longer flame but a thinner flame brush, stretching over the range 2.1 < *z*/*d* < 2.8. Moreover, $\widetilde {Y}_{\text {OH}}$ in the burnt gases, downstream of the flame is lower than in the pilot stream, whilst this trend is not observed in the OH PLIF signal intensity. The simulation results also show that a significant proportion of fuel is not burned and the presence of this fuel in the hot combustion products leads to the production of CH_2_O downstream of the flame. The leaner flame (Fig. 6, top) with an overall equivalence ratio of *ϕ* = 0.62 is longer and experiments showed that the mean flame brush is present in the range 2.5 < *z*/*d* < 3.5 on the burner axis. This flame length is well predicted by the simulation. Moreover, the simulation also predicts a lower $\widetilde {Y}_{\text {OH}}$ in the main flow compared to the pilot stream. Indeed, this behaviour is also found in the OH PLIF signal of the leaner flame. However, this feature is less pronounced in this case and the simulations, unexpectedly, predict that the combustion products of the leaner flame contain more $\widetilde {Y}_{\text {OH}}$ than in the case of the richer flame. Since the pre-vaporised fraction of fuel is different in both cases, this feature can be directly related to the droplet terms in the transport equations of the conditioning variables, which will be discussed next.

**Fig. 7:**
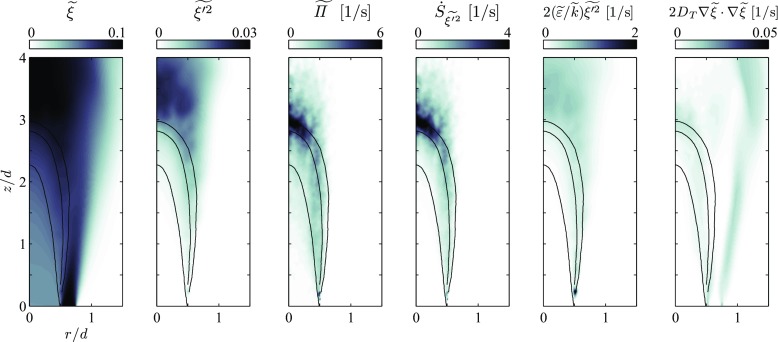
Fields of $\widetilde {\xi }$ and $\widetilde {\xi ^{\prime 2}}$ as well as source/sink terms of their Favre-averaged transport equations for the flame with *ϕ* = 0.82. $\dot {S}_{\widetilde {\xi ^{\prime 2}}}$ represents the complete droplet source in the mixture fraction variance equation and $2(\widetilde {\varepsilon }/\widetilde {k}) \widetilde {\xi ^{\prime 2}}$ is the scalar dissipation rate term. Isolines of $\widetilde {c}= 0.1$, 0.5 and 0.9 mark the position of the mean flame brush

In the following, some information on the relative magnitude of the various terms in the transport equations of $\widetilde {\xi }$, $\widetilde {\xi ^{\prime 2}}$, $\widetilde {c}$, $\widetilde {c^{\prime 2}}$ and the DCMC equation is given. Figure 7 shows the field of the mean mixture fraction and its variance for the richer flame with *ϕ* = 0.82. The mean mixture fractions set at the inlet, one nozzle diameter upstream of the burner exit, is below the lean flammability limit of an ethanol air mixture (*ξ*_lean_ ≈ 0.05) to $\widetilde {\xi }$. Upstream of the flame the mean temperature is low and the evaporation rate $\widetilde {\varPi }$ is low. Nevertheless $\widetilde {\xi }$ increases along the burner axis, first slowly to $\widetilde {\xi }\approx 0.048$ at *z*/*d* = 1 and then faster, reaching 0.59 at *z*/*d* = 2 in the preheat zone of the flame. Even though the evaporation rate is highest in the range of $0.5<\widetilde {c}<0.9$, some droplets still exist downstream of the flame brush and $\widetilde {\varPi }$ takes significant values until *z*/*d* = 3.5. Droplet evaporation is also the dominant source in the mixture fraction variance equation (14) and the production term due to mean mixture fraction gradients is negligible in the flame investigated in this work. In contrast to $\widetilde {\varPi }$, the mixture fraction variance source term is very small upstream of the $\widetilde {c}= 0.1$ isoline because the droplets evaporating in this region have a low *T*_*d*_ and thus *ξ*_*s*_ is not much larger than $\widetilde {\xi }$. Consequently, $\widetilde {\xi ^{\prime 2}}$ rises significantly in the region of the mean flame brush. In particular, this increase of $\widetilde {\xi ^{\prime 2}}$ is not directly counterbalanced by the scalar dissipation rate term, since a model for passive scalar mixing [29], was used due to the lack of alternatives. Even though the scalar dissipation rate is globally of the same order of magnitude as the droplet source of $\widetilde {\xi ^{\prime 2}}$, we note that $\widetilde {\xi ^{\prime 2}}$ is locally over-predicted. In particular, this is the case in the core of the main flow for 2.5 < *z*/*d* < 3.5, where the production of variance outweighs its destruction (Fig. 7).

This high level of mixture fraction variance in the post flame region of the richer flame explains low levels of $\widetilde {Y}_{\text {OH}}$ in the main flow of the richer flame, compared to the pilot stream (Fig. 6). For high $\widetilde {\xi ^{\prime 2}}$ a broad *β*-pdf is presumed and the probability of finding flammable mixture that can react to form OH is low. This connection is also demonstrated by the unexpected fact that the simulations show higher $\widetilde {Y}_{\text {OH}}$ for the leaner flame. Since the mass fraction of prevaporised fuel is $\widetilde {\xi }= 0.04$ for both flames, in the lean case two thirds of fuel mass are fully premixed. Hence, evaporation produces lower levels of $\widetilde {\xi ^{\prime 2}}$ in the lean case leading to higher $\widetilde {Y}_{\text {OH}}$. For the same reason $\bar {\rho }$ is over-predicted leading to an under-prediction of $\widetilde {u}_{z}$ at downstream locations.

**Fig. 8:**
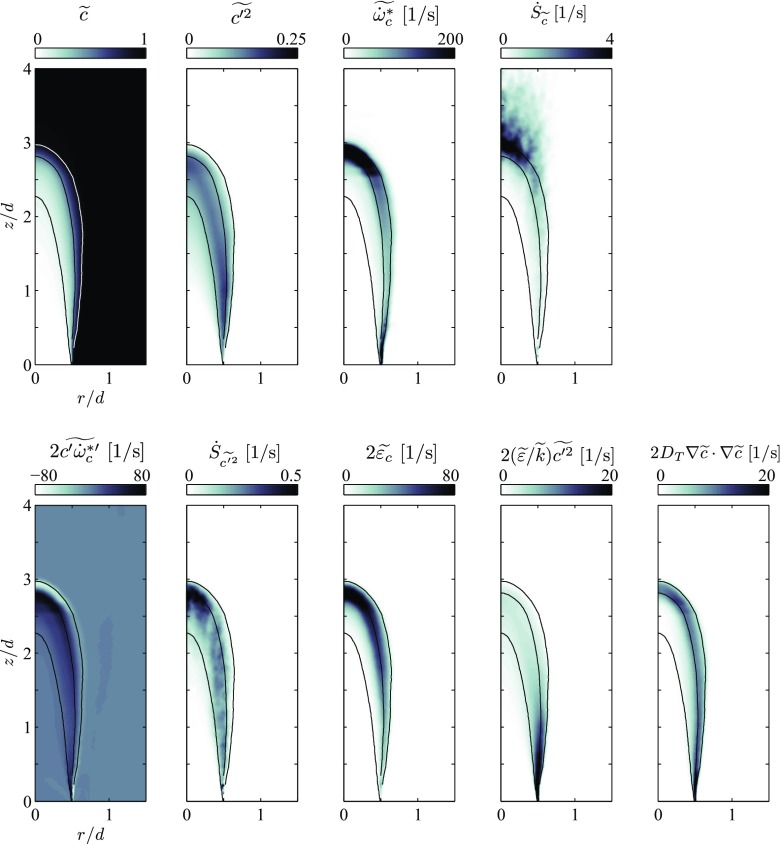
Fields of $\widetilde {c}$ and $\widetilde {c^{\prime 2}}$ as well as source/sink terms of their Favre-averaged transport equations for the flame with *ϕ* = 0.82. $\dot {S}_{\widetilde {c}}$ and $\dot {S}_{\widetilde {c^{\prime 2}}}$ represent the complete droplet sources of mean and variance of the reaction progress variable respectively. $\widetilde {\varepsilon }_{c}$ is the scalar dissipation rate computed using the model by Kolla et al. [30]. Isolines of $\widetilde {c}= 0.1$, 0.5 and 0.9 mark the position of the mean flame brush

**Fig. 9:**
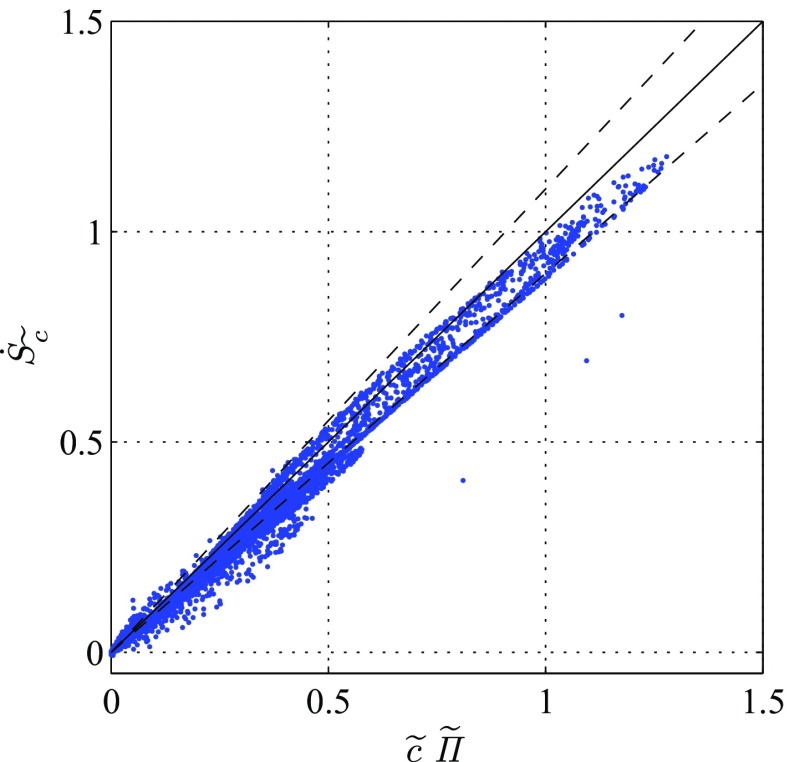
Scatter plot of $\widetilde {c}\widetilde {\varPi }$ versus $\dot {S}_{\widetilde {c}}$. The straight line represents equality; the dashed lines are for + 10% and − 10%. Simulation data from the richer flame with *ϕ* = 0.82

Fields of $\widetilde {c}$ and $\widetilde {c^{\prime^2}}$ are shown in Fig. 8. In addition to the mean reaction source term, the $\widetilde {c}$-equation (8) also contains a droplet source term, which is computed as detailed in Eq. 22. As previously discussed, this source term is zero as long as the droplets evaporate in unburned mixture. On a global scale $\dot {S}_{\widetilde {c}}$ is also two orders of magnitude lower than the apparent reaction rate $\widetilde {\dot {\omega }_{c}^{*}}$ and, thus, it only has a small effect on the shape of the flame brush. The same applies to the total evaporation variance source term $\dot {S}_{\widetilde {c^{\prime 2}}}$ compared to $\widetilde {c^{\prime }\dot {\omega }_{c}^{*\prime }}$, such that the effect of evaporation on the $\widetilde {c^{\prime 2}}$-equation is negligible in the present case. In contrast, the $\dot {S}_{\widetilde {c}}$ plays an important role in the region downstream of the flame, where it counter-balances a large fraction of the decrease in $\widetilde {c}$, otherwise caused by the evaporative mass source acting on the mean density. For this purpose, a simplified model for this term can be proposed by comparing $\dot {S}_{\widetilde {c}}$ to $\widetilde {c}\widetilde {\varPi }$. Figure 9 shows that instead of using Eq. 22, a simplified model,

26 $$\begin{array}{@{}rcl@{}} \dot{S}_{\widetilde{c}} = \widetilde{\mathscr{C}\varPi} + \widetilde{c\varPi} \approx \widetilde{c}\widetilde{\varPi} \end{array} $$

can be used for this purpose.

This leaves the reaction source $\widetilde {c^{\prime }\dot {\omega }_{c}^{*\prime }}$ and the production due to mean progress variable gradients as the main source terms in the $\widetilde {c^{\prime 2}}$-equation. Note that $\widetilde {c^{\prime }\dot {\omega }_{c}^{*\prime }}$ locally also takes the role of an important variance sink term as the reaction reaches completion. On the side of the sink terms Fig. 8 shows a comparison of the mean scalar dissipation rate $\widetilde {\varepsilon _{c}}$, as computed in the present work using the model by Kolla et al. [30], and the model for a passive scalar [29] applied to the reaction progress variable. The latter takes the largest values in the thinnest part of the reacting shear layer that forms between the main flow and the pilot stream, but takes much smaller values than $\widetilde {\varepsilon _{c}}$ in the region where the flame closes. Indeed, in the present simulations it was not possible to stabilise a flame using $2(\widetilde {\varepsilon }/\widetilde {k}) \widetilde {c^{\prime 2}}$ to model the scalar dissipation rate, such that the model by Kolla et al. [30] was employed.

## Conclusions

The CMC framework was used to build a new model for spray flames, which may feature characteristics of both premixed and non-premixed combustion. The DCMC equation for spray flames, conditioned on mixture fraction and reaction progress variable, was presented in this work. In the derivation, spray evaporation terms in the transport equations of the reactive scalar and both conditioning variables, including *c*, were explicitly considered, which added new terms to the DCMC equation.

In a preliminary application, the model was used to simulate the behaviour of a piloted ethanol spray flame with significant pre-vaporisation and strong premixing at two different, lean conditions. For this purpose, a set of sub-models was proposed to provide closure for the DCMC model and an operator splitting procedure was suggested to solve the model equation. The velocity field and the droplet distributions showed good agreement with experimental data and the flame shape prediction was promising in revealing the experimental trend due to overall equivalence ratio.

Using the conditional moments of the reactive scalars, available from DCMC, the unclosed spray terms in the Favre-averaged transport equations of the conditioning variables were modelled. Droplet evaporation was the dominant source term in the mixture fraction variance equation and had a large effect on the result, which had also been reported in previous studies. At the same time, closure of the mixture fraction scalar dissipation rate with a passive scalar mixing model seemed to be inadequate and main differences between simulation and experiment could be related to this imbalance of variance source and sink terms. The spray terms in the reaction progress variables equations were small compared to the reaction source terms and, thus, had little effect on the shape and width of the mean flame brush. This suggests that the effect of droplet evaporation can be neglected in the $\widetilde {c^{\prime 2}}$-equation. However, the evaporation source term should be included in the mean progress variable equation, but it can be approximated with a simple model.

## Appendix

### *Q*_*h*_-equation

The derivation of the local instantaneous transport equation of the enthalpy is detailed in the text book by Poinsot and Veynante [42]. Using the Kataoka model [18] leads to the *h*-equation for a two-phase flow,

27 $$\begin{array}{@{}rcl@{}} \frac{\partial \theta \rho h}{\partial t} + \text{div}(\theta \rho h \mathbf{u} ) &= & \text{div}(\theta \rho \lambda \nabla T) +\sum\limits_{\alpha} \text{div}(\theta\rho h_{\alpha} D_{\alpha}\nabla Y_{\alpha}) \\ &&+\theta \frac{\partial p}{\partial t} +\theta \mathbf{u}\cdot\nabla p + \theta \dot{q} +\theta \boldsymbol{\tau} : (\nabla\mathbf{u})^{\intercal} +\sum\limits_{\alpha}\rho \mathbf{f}_{\alpha}\cdot Y_{\alpha}\mathbf{V}_{\alpha}\\ &&-\nabla\theta\cdot\lambda\nabla T +\sum\limits_{\alpha}\rho h_{\alpha} Y_{\alpha}\hat{V}_{\alpha} + \rho h\varPi \end{array} $$

where $\dot {q}$ is a heat source, for instance due to a spark or radiation and the third line represents the source term due to droplet evaporation.

The DCMC equation for the conditional enthalpy *Q*_*h*_ is as follows,

28 $$\begin{array}{@{}rcl@{}} \frac{\partial Q_{h}}{\partial t} & + & \langle \mathbf{u} | \eta, \zeta \rangle \cdot \nabla Q_{h} = - \frac{1}{\bar{\theta}\bar{\rho}\widetilde{p}} \text{div} \left( \bar{\theta}\bar{\rho}\widetilde{p} \langle \mathbf{u}^{\prime\prime} h^{\prime\prime} | \eta, \zeta \rangle \right) \\ &+& \frac{1}{\bar{\rho}}\langle \frac{\partial p}{\partial t}|\eta,\zeta\rangle + \frac{1}{\bar{\rho}}\langle \mathbf{u}\cdot\nabla p|\eta,\zeta\rangle + \frac{1}{\bar{\rho}}\langle \boldsymbol{\tau} : (\nabla\mathbf{u})^{\intercal}|\eta,\zeta\rangle + \frac{1}{\bar{\rho}}\langle \dot{q}|\eta,\zeta\rangle\\ &+&\langle \text{Le}_{\xi} N_{\xi}|\eta,\zeta\rangle \frac{\partial^{2} Q_{h}}{\partial \eta^{2}} + \langle \text{Le}_{c} N_{c}|\eta,\zeta\rangle \frac{\partial^{2} Q_{h}}{\partial \zeta^{2}} \\ &+& \langle (\text{Le}_{\xi} + \text{Le}_{c}) N_{\xi c}|\eta,\zeta\rangle \frac{\partial^{2} Q_{h}}{\partial \eta \partial \zeta} - \langle \dot{\omega}_{c}^{*} | \eta, \zeta \rangle \frac{\partial Q_{h}}{\partial \zeta} \\ &+& \langle h\varPi|\eta,\zeta\rangle + \sum\limits_{\alpha}^{N_{\alpha}} \frac{\langle h_{\alpha} (\delta_{\alpha}-h) \varPi | \eta,\zeta \rangle}{\bar{\theta}} - \frac{\langle \nabla\theta\cdot\lambda\nabla T|\eta,\zeta\rangle}{\bar{\theta}\bar{\rho}}\\ &-& \left[ \left( 1 - \eta \right) \frac{\partial Q_{h}}{\partial \eta} + \mathscr{C}(\eta,\zeta) \frac{\partial Q_{h}}{\partial \zeta} \right] \frac{\langle \varPi | \eta,\zeta \rangle}{\bar{\theta}} \\ &-& \frac{1}{\bar{\theta}\bar{\rho} \widetilde{p}} \frac{\partial \ \bar{\rho}\widetilde{p} (1-\eta) \langle h^{\prime\prime} \varPi^{\prime\prime} |\eta,\zeta\rangle}{\partial\eta} - \frac{1}{\bar{\theta}\bar{\rho} \widetilde{p}} \frac{\partial \ \bar{\rho}\widetilde{p} \mathscr{C}(\eta,\zeta) \langle h^{\prime\prime} \varPi^{\prime\prime} |\eta,\zeta\rangle}{\partial\zeta} \\ &-& \frac{1}{\bar{\theta}\bar{\rho} \widetilde{p}} \frac{\partial \ \bar{\theta}\bar{\rho}\widetilde{p} \langle h^{\prime\prime} \dot{\omega}_{c}^{*\prime\prime} |\eta,\zeta\rangle}{\partial\zeta} + \mathcal{D}_{Qh} + \mathcal{D}_{Dh} \end{array} $$

In Eq. 28, $\mathcal {D}_{Qh}$ represents the differential diffusion term,

29 $$\begin{array}{@{}rcl@{}} \mathcal{D}_{Qh}& = & \frac{1}{\bar{\theta}\bar{\rho}\widetilde{p}} \frac{\partial \ \bar{\theta}\bar{\rho}\widetilde{p} \langle (\text{Le}_{\xi}-1)D_{\xi} \nabla \xi \cdot \nabla \xi |\eta, \zeta \rangle }{\partial \eta} \frac{\partial Q_{h}}{\partial \eta}\\ &&+ \frac{1}{\bar{\theta}\bar{\rho}\widetilde{p}} \frac{\partial \ \bar{\theta}\bar{\rho}\widetilde{p} \langle (\text{Le}_{\xi} - 1)D_{\xi} \nabla \xi \cdot \nabla c | \eta, \zeta \rangle }{\partial \zeta} \frac{\partial Q_{h}}{\partial \eta}\\ &&+ \frac{1}{\bar{\theta}\bar{\rho}\widetilde{p}} \frac{\partial \ \bar{\theta}\bar{\rho}\widetilde{p} \langle (\text{Le}_{c} - 1)D_{c} \nabla \xi \cdot \nabla c |\eta, \zeta \rangle }{\partial \eta} \frac{\partial Q_{h}}{\partial \zeta}\\ &&+ \frac{1}{\bar{\theta}\bar{\rho}\widetilde{p}} \frac{\partial \ \bar{\theta}\bar{\rho}\widetilde{p} \langle (\text{Le}_{c} - 1)D_{c} \nabla c \cdot \nabla c |\eta, \zeta \rangle }{\partial \zeta} \frac{\partial Q_{h}}{\partial \zeta}\\ &&- \frac{1}{\bar{\theta}\bar{\rho}\widetilde{p}} \sum\limits_{\alpha}^{N_{\alpha}} \frac{\partial}{\partial \eta} \left( \bar{\theta}\bar{\rho}\widetilde{p} \langle h_{\alpha} (\text{Le}_{\alpha}-1) D_{\alpha} \nabla \xi \cdot \nabla \xi |\eta, \zeta \rangle \frac{\partial Q_{\alpha}}{\partial \eta} \right) \\ && - \frac{1}{\bar{\theta}\bar{\rho}\widetilde{p}} \sum\limits_{\alpha}^{N_{\alpha}} \frac{\partial}{\partial \eta} \left( \bar{\theta}\bar{\rho}\widetilde{p} \langle h_{\alpha} (\text{Le}_{\alpha}-1) D_{\alpha} \nabla \xi \cdot \nabla c |\eta, \zeta \rangle \frac{\partial Q_{\alpha}}{\partial \zeta} \right) \\ && - \frac{1}{\bar{\theta}\bar{\rho}\widetilde{p}} \sum\limits_{\alpha}^{N_{\alpha}} \frac{\partial}{\partial \zeta} \left( \bar{\theta}\bar{\rho}\widetilde{p} \langle h_{\alpha} (\text{Le}_{\alpha}-1) D_{\alpha} \nabla \xi \cdot \nabla c |\eta, \zeta \rangle \frac{\partial Q_{\alpha}}{\partial \eta} \right) \\ && - \frac{1}{\bar{\theta}\bar{\rho}\widetilde{p}} \sum\limits_{\alpha}^{N_{\alpha}} \frac{\partial}{\partial \zeta} \left( \bar{\theta}\bar{\rho}\widetilde{p} \langle h_{\alpha} (\text{Le}_{\alpha}-1) D_{\alpha} \nabla c \cdot \nabla c |\eta, \zeta \rangle \frac{\partial Q_{\alpha}}{\partial \zeta} \right) \end{array} $$

Without the high Reynolds number assumptions, the *Q*_*h*_-equation also contains molecular diffusion terms $\mathcal {D}_{Dh}$.

30 $$\begin{array}{@{}rcl@{}} \mathcal{D}_{Dh} & = & \frac{1}{\bar{\theta}\bar{\rho}\widetilde{p}} \text{div}\left( \bar{\theta}\bar{\rho}\widetilde{p}\ \langle\text{Le}_{\xi} D_{\xi} \nabla h |\eta,\zeta\rangle \right)\\ && - \frac{1}{\bar{\theta}\bar{\rho}\widetilde{p}} \sum\limits_{\alpha}^{N_{\alpha}} \text{div} \left( \bar{\theta}\bar{\rho}\widetilde{p} \langle h_{\alpha} (\text{Le}_{\alpha}-1) D_{\alpha} \nabla Y_{\alpha} |\eta, \zeta \rangle \right)\\ && - \frac{1}{\bar{\theta}\bar{\rho}\widetilde{p}} \frac{\partial \ \text{div}(\bar{\theta}\bar{\rho}\widetilde{p} \langle h D_{\xi} \nabla \xi |\eta, \zeta \rangle ) }{\partial \eta} - \frac{1}{\bar{\theta}\bar{\rho}\widetilde{p}} \frac{\partial \ \text{div}(\bar{\theta}\bar{\rho}\widetilde{p} \langle h D_{c} \nabla c |\eta, \zeta \rangle ) }{\partial \zeta}\\ && + \frac{Q_{h}}{\bar{\theta}\bar{\rho}\widetilde{p}} \frac{\partial \ \text{div}(\bar{\theta}\bar{\rho}\widetilde{p} \langle D_{\xi} \nabla \xi |\eta, \zeta \rangle ) }{\partial \eta} + \frac{Q_{h}}{\bar{\theta}\bar{\rho}\widetilde{p}} \frac{\partial \ \text{div}(\bar{\theta}\bar{\rho}\widetilde{p} \langle D_{c} \nabla c |\eta, \zeta \rangle ) }{\partial \zeta}\\ && + \frac{1}{\bar{\theta}\bar{\rho}\widetilde{p}} \frac{\partial \ \bar{\theta}\bar{\rho}\widetilde{p} \langle (\text{Le}_{\xi}+ 1)D_{\xi} \nabla \xi |\eta, \zeta \rangle \cdot \nabla Q_{h} }{\partial \eta}\\ &&+ \frac{1}{\bar{\theta}\bar{\rho}\widetilde{p}} \frac{\partial \ \bar{\theta}\bar{\rho}\widetilde{p} \langle (\text{Le}_{c}+ 1)D_{c} \nabla c |\eta, \zeta \rangle \cdot \nabla Q_{h} }{\partial \zeta} \\ && - \frac{1}{\bar{\theta}\bar{\rho}\widetilde{p}} \sum\limits_{\alpha}^{N_{\alpha}} \frac{\partial \bar{\theta}\bar{\rho}\widetilde{p} \langle h_{\alpha} (\text{Le}_{\alpha}-1) D_{\alpha} \nabla\xi |\eta, \zeta \rangle \cdot\nabla Q_{\alpha}}{\partial\eta} \\ && - \frac{1}{\bar{\theta}\bar{\rho}\widetilde{p}} \sum\limits_{\alpha}^{N_{\alpha}} \frac{\partial \bar{\theta}\bar{\rho}\widetilde{p} \langle h_{\alpha} (\text{Le}_{\alpha}-1) D_{\alpha} \nabla c |\eta, \zeta \rangle \cdot\nabla Q_{\alpha}}{\partial\zeta} \end{array} $$

The analogue molecular diffusion term, as it would appear in the *Q*_*α*_-equation is shown below.

### Molecular diffusion term of the *Q*_*α*_-equation

In Eq. 10 high Reynolds number was assumed and, thus, molecular transport was neglected in the derivation. Without this assumption the following term would appear, which is analogue to the $\mathcal {D}_{Dh}$-term in the *Q*_*h*_-equation (30).

31 $$\begin{array}{@{}rcl@{}} \mathcal{D}_{D_{\alpha}} & = & \frac{1}{\bar{\theta}\bar{\rho}\widetilde{p}} \text{div}\left( \bar{\theta}\bar{\rho}\widetilde{p}\ \langle D_{\alpha} \nabla Y_{\alpha} |\eta,\zeta\rangle \right) \\ && - \frac{1}{\bar{\theta}\bar{\rho}\widetilde{p}} \frac{\partial \ \text{div}(\bar{\theta}\bar{\rho}\widetilde{p} \langle Y_{\alpha} D_{\xi} \nabla\xi |\eta,\zeta \rangle ) }{\partial \eta} - \frac{1}{\bar{\theta}\bar{\rho}\widetilde{p}} \frac{\partial \ \text{div}(\bar{\theta}\bar{\rho}\widetilde{p} \langle Y_{\alpha} D_{c} \nabla c |\eta, \zeta \rangle ) }{\partial \zeta}\\ && + \frac{Q_{\alpha}}{\bar{\theta}\bar{\rho}\widetilde{p}} \frac{\partial \ \text{div}(\bar{\theta}\bar{\rho}\widetilde{p} \langle D_{\xi} \nabla \xi |\eta, \zeta \rangle ) }{\partial\eta} + \frac{Q_{\alpha}}{\bar{\theta}\bar{\rho}\widetilde{p}} \frac{\partial \ \text{div}(\bar{\theta}\bar{\rho}\widetilde{p} \langle D_{c} \nabla c |\eta, \zeta \rangle ) }{\partial\zeta}\\ && + \frac{1}{\bar{\theta}\bar{\rho}\widetilde{p}} \frac{\partial \ \bar{\theta}\bar{\rho}\widetilde{p} \langle (D_{\alpha}+D_{\xi}) \nabla \xi |\eta, \zeta \rangle \cdot \nabla Q_{\alpha} }{\partial \eta}\\ &&+ \frac{1}{\bar{\theta}\bar{\rho}\widetilde{p}} \frac{\partial \ \bar{\theta}\bar{\rho}\widetilde{p} \langle (D_{\alpha}+D_{c}) \nabla c |\eta, \zeta \rangle \cdot \nabla Q_{\alpha} }{\partial \zeta} \end{array} $$
